# Severe Kawasaki disease in a 3-month-old patient: a case report

**DOI:** 10.1186/1756-0500-6-500

**Published:** 2013-12-02

**Authors:** Salvatore Leonardi, Patrizia Barone, Giacomo Gravina, Giuseppe Fabio Parisi, Valeria Di Stefano, Pietro Sciacca, Mario La Rosa

**Affiliations:** 1 Unit of Broncho-Pneumology and Cystic Fibrosis, Department of Medical and Pediatric Science, University of Catania, Via Santa Sofia 78, Catania 95123, Italy

**Keywords:** Kawasaki disease, Infant, Coronary artery aneurysms, Therapy

## Abstract

**Background:**

Kawasaki disease is a multi-system vasculitis which usually occurs in children under 5 years of age. In infants under three months of age, it is very rare and usually associated with a high incidence of incomplete or atypical forms, often unresponsive to treatment. This condition increases the risk of cardiovascular complications such as coronary artery aneurysms.

**Case presentation:**

We describe a 3-month-old infant who developed early and severe aneurysms in three coronary arteries despite a timely administration of intravenous immunoglobulins, followed by three days of intravenous methylprednisolone.

**Conclusion:**

This case report underlines that the development of coronary artery aneurysm correlates with a delayed diagnosis and treatment, incomplete or atypical forms of the disease, and additionally the severity of clinical presentation, especially in cases of very young infants below 3 months of age. Our case is notable because of the very young age of the patient, the severity of clinical presentation with an early development of coronary artery aneurysms and the unresponsiveness to the therapy.

## Background

Kawasaki disease (KD) is an acute multisystem necrotizing vasculitis of medium and small-size vessels of unknown etiology [1], usually occurring in infants and children under 5 years [2,3]. KD was described for the first time in 1967 by Tomisaku Kawasaki and it was named “mucocutaneous lymph-node syndrome” [4]. Today it is known for its occurrence in small epidemics especially within closed communities and for its higher incidence in Asian populations [5].

The diagnosis of classic KD is based on the simultaneous presence of high fever for 5 or more days with at least four of the remaining five symptoms (bilateral conjunctival hyperemia, ulcerations of the lips and inflammation of the oral cavity, polymorphous rash, edema and desquamation of the extremities and cervical lymphadenopathy) or fever associated with less than 4 of the diagnostic criteria and echocardiographic abnormalities of the coronary arteries. Coronary artery aneurysms or ectasias may develop in 25-30% of untreated children and may even lead to ischemic heart disease, myocardial infarction (MI) or sudden death [4,6].

In the acute phase, the aim of treatment is to reduce the inflammation in the coronary artery wall and to prevent coronary thrombosis whereas the long-term therapy, especially in patients with coronary ectasias or aneuryms, is to prevent myocardial damage [6].

Presently, KD continues to be a disease with several problems [3]. The main difficulties for clinicians are how to perform a timely diagnosis, how to prevent cardiovascular complications, and how to treat refractory forms. Refractory forms have been increasing markedly and both young age of the patient and a delay in starting the treatment seem to be major risk factors [7-9].

We describe a case of a 3-month-old male infant with KD who developed severe coronary artery lesions despite an early diagnosis and a timely administration of intravenous immunoglobulin (IVIG).

## Case presentation

A 3-month-old Caucasian male infant was admitted to our department because of 24 hours of high-grade persistent fever (T 39.5°C) not relieved by acetaminophen.

**Figure 1:**
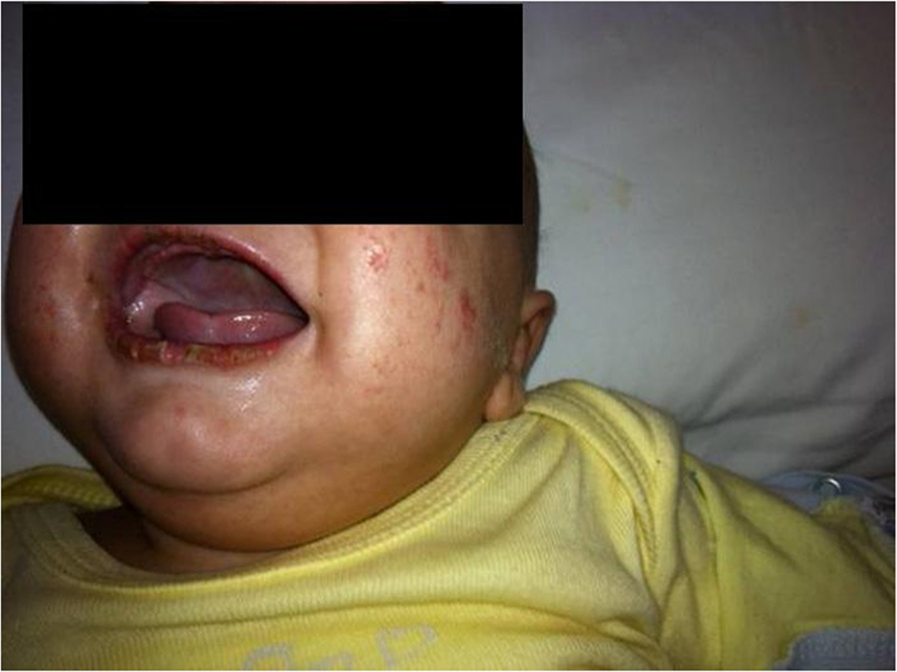
**Patient’s face.** Note fissures of the lips, inflammation of the oral cavity and polymorphous rash.

He was the second-born of non-consanguineous parents, after 36 weeks gestation after an unremarkable pregnancy. Birth weight was 3015 g. On admission, his general condition was poor because of high fever (T 39.5°C), tachycardia and tachypnea. On physical examination, he presented with generalized edema and non-palpable peripheral lymph-nodes. Muscle tone was normal and lungs and heart examination was unremarkable. Pharynx was hyperemic. Abdomen examination was normal: liver and spleen were within normal limits. Meningeal signs were absent but the patient was very irritable (Figure 1).

At the admission, laboratory test showed normocytic anemia (hemoglobin 9 g/dL, red blood cells 3,180,000/mm^3^, mean corpuscular volume 80 fl), neutrophilic leucocytosis (white blood cells 28,300/mm^3^, neutrophils 69%) with a normal platelet count (200,000/mm^3^). Laboratory investigations also showed elevated gamma-glutamyltransferase (52 U/L), hyperbilirubinemia (2.98 mg/dL), hypoalbuminemia (2.5 g/dL), hypoproteinemia (4.3 g/dL), hyponatremia (128 mEq/L); transaminase levels were normal (aspartate aminotransferase 45 IU/L, alanine aminotransferase 40 IU/L). C-reactive protein (CRP) confirmed the significant state of inflammation (12.39 mg/dL).

**Figure 2:**
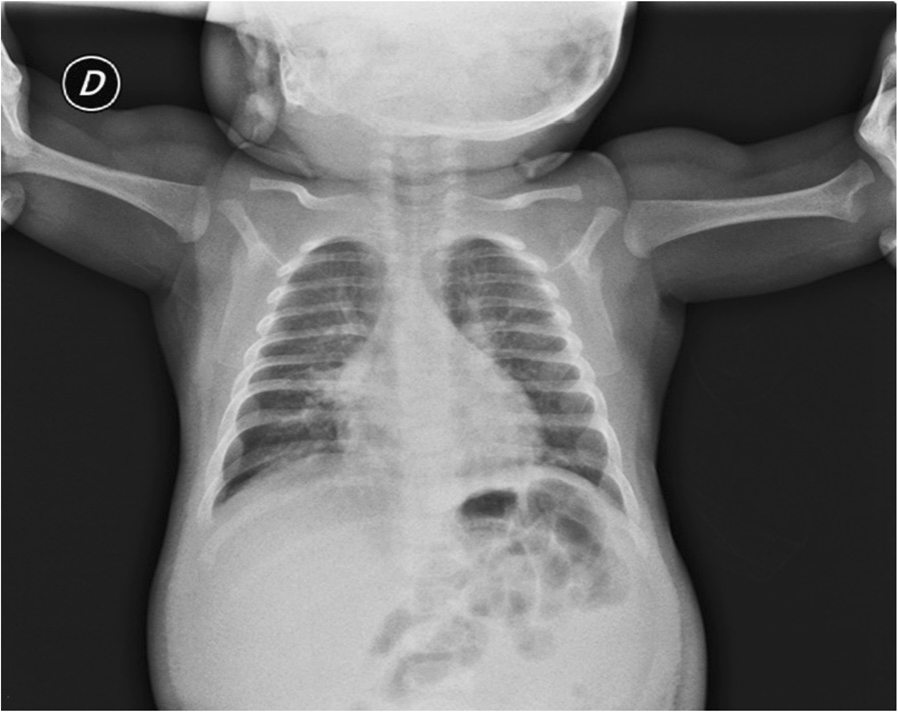
**Chest radiography.** Generalized increased translucency of the thorax. The heart size was within normal limits.

Chest radiography showed a generalized increased translucency of the thorax. The heart size was within normal limits (Figure 2).

Abdominal ultrasound revealed slight hepatosplenomegaly and mild peritoneal effusion.

Right coronary artery (RCA) on echocardiography resulted to be within the maximum limits of normality with mild hyperechogenicity of the wall. A small pericardial effusion was detected too.

The patient was initially treated with intravenous antibiotic therapy (ceftriaxone) but, because of the persistence of the fever we suspected KD, we started the first dose of IVIG (2 g/kg in a single infusion) and replaced acetaminophen with ibuprofen.

**Figure 3:**
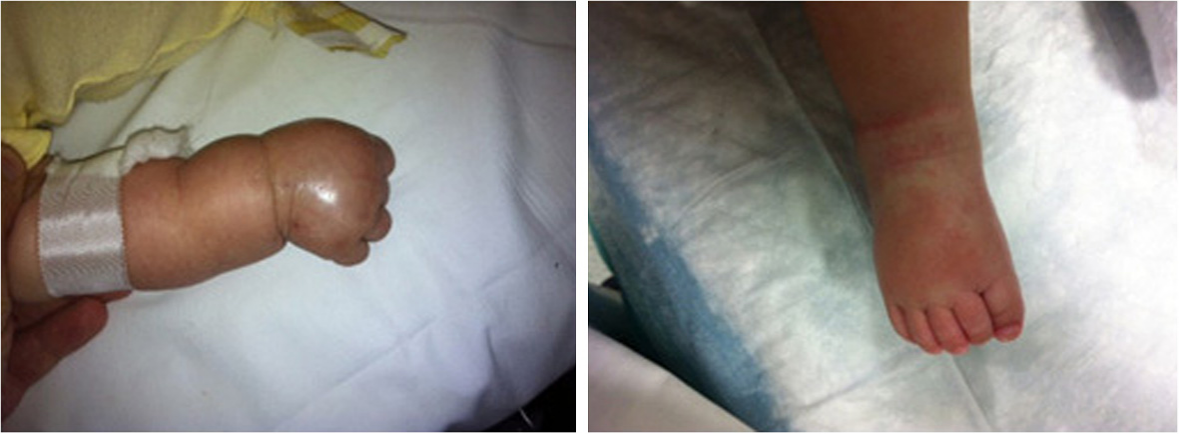
**Patient’s limbs.** Painful, brawny edema of the dorsa of the hand and foot.

Nevertheless, the child continued to be febrile and a generalized rash starting from the trunk and conjunctivitis appeared. In addition, the hands and feet became swollen (Figure 3).

Echocardiography examination performed after 48 hours from the first dose of IVIG showed a dilatation of RCA (0.25 cm).

Due to the persistence of fever we performed a second dose of IVIG.

**Figure 4:**
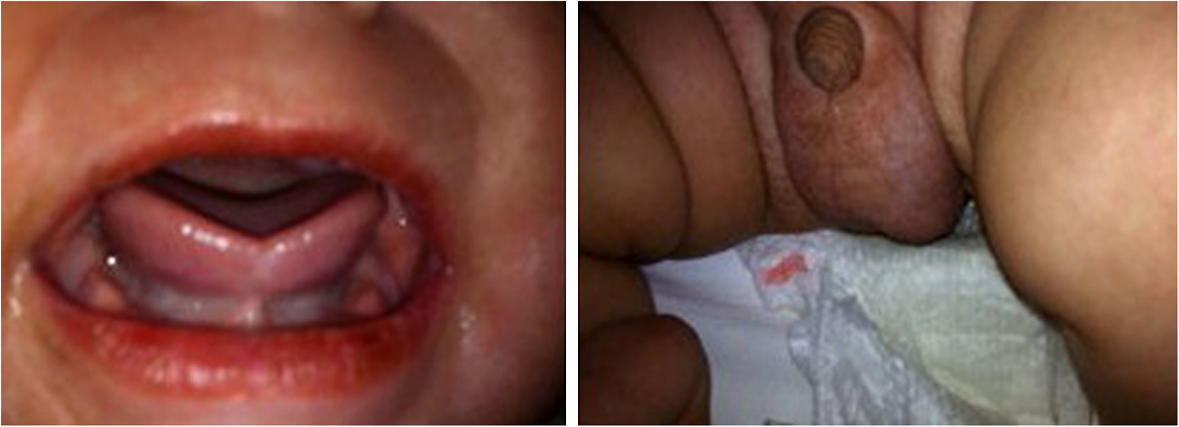
**Patient’s mouth and genitals.** Fissuring of the lips and hydrocele.

**Figure 5:**
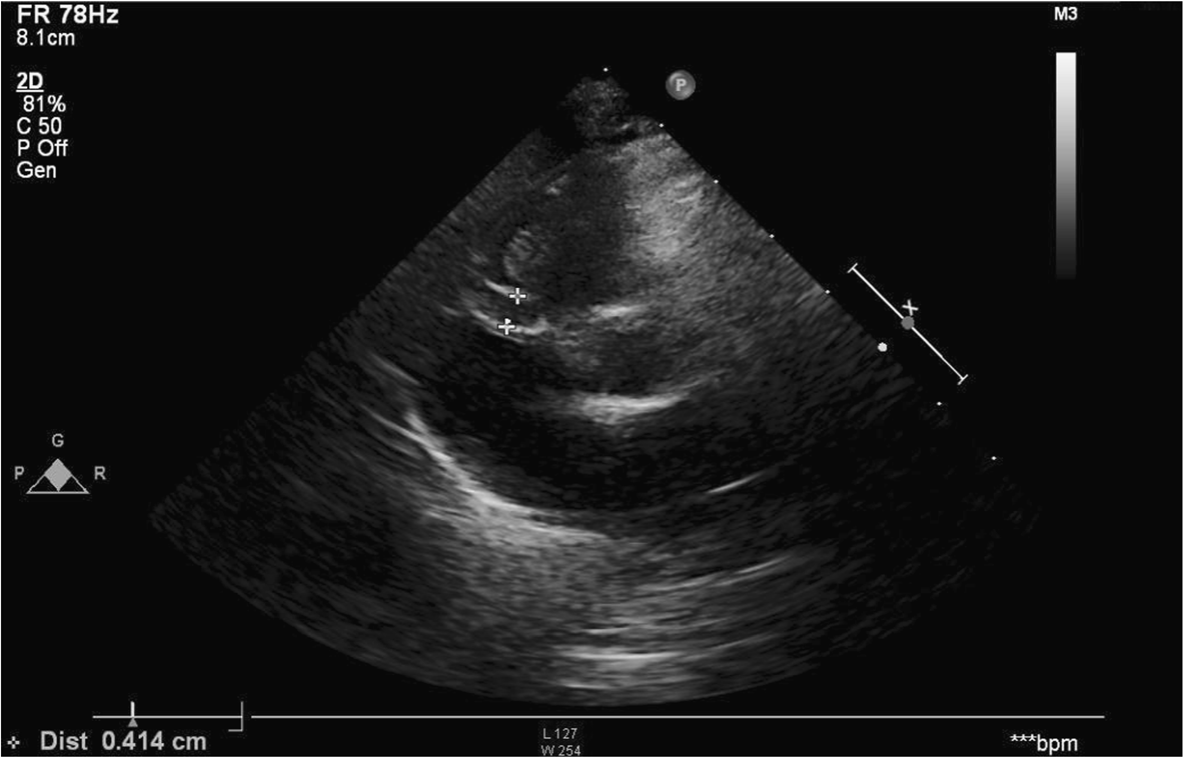
**Echocardiography.** After second treatment with intravenous immunoglobulin the patient developed ectasia of left coronary artery and a worsening of the dilatation of right coronary artery.

After this treatment the patient developed fissuring of the lips, hydrocele (Figure 4), ectasia of left coronary artery (LCA) and a worsening of the dilatation of RCA (RCA: 0.45 cm; LCA: 0.32 cm) (Figure 5).

On the 10th day of illness, because of persistent fever, we performed a bone marrow aspiration which showed no cellular abnormalities and we started the administration of intravenous methylprednisolone (IVMP, 30 mg/kg/day) for 3 consecutive days together with high-dose of acetylsalicylic acid (80 mg/kg/day).

Although this treatment, laboratory tests showed worsening of thrombocytosis (from 143,000/mm3 to 298,000/mm3), neutrophilic leukocytosis, anemia with reticulocytosis, a further increase of CRP value and a persistence of the fever.

Because of the marked increase of transaminase levels (aspartate aminotransferase 230 IU/L, alanine aminotransferase 427 IU/L), a week later, the dose of aspirin was slightly reduced (60 mg/kg/day).

Following the IVMP therapy, we decided to continue steroid therapy by oral prednisone at dosage of 2 mg/kg/day and, after the improvement of the clinical condition, we reduced the dosage at 1mg/kg/day two weeks later.

**Figure 6:**
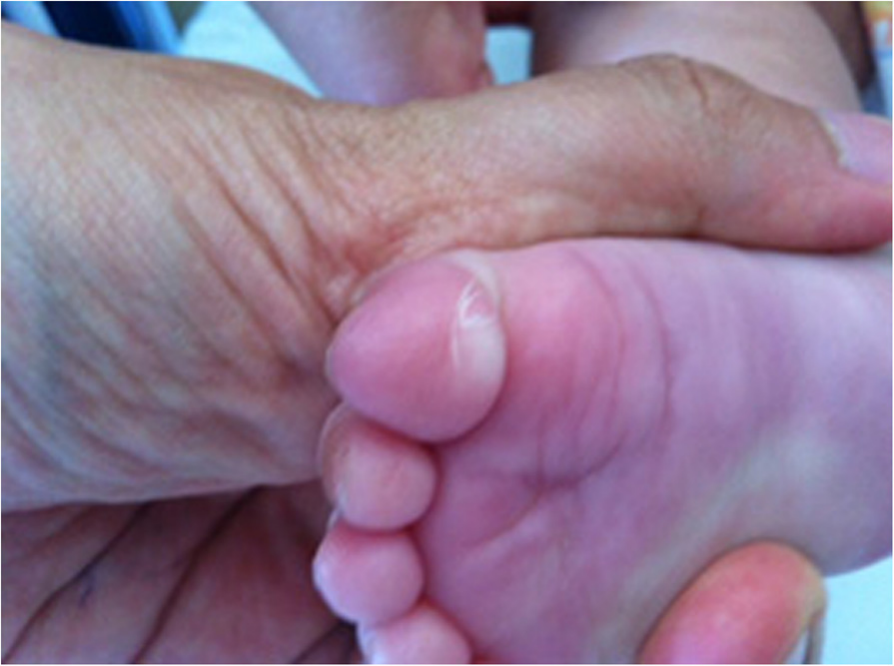
Periungueal desquamation.

Periungueal desquamation began on day fifteen and lasted for a week (Figure 6). Although fever disappeared 19 days after onset of the illness and inflammatory indexes turned into normal values, platelet count remained high (Table 1) and for this reason we reduced the dosage of acetylsalicylic acid at 5 mg/kg/day for its anti-aggregant effects.

**Table 1:** Laboratory findings of the patient

**Day**	**Hemoglobin (g/dL)**	**Platelets (/mm**^**3**^**)**	**White blood cells (/mm**^**3**^**)**	**Neutrophils (%)**	**C-reactive protein (mg/dL)**	**Albumin (g/dL)**	**Sodium (mMol/L)**
**1**	9	200,000	28,300	69	12.39		
**3**	8.1	335,000	15,320	83	24	2.5	130
**4**	7.7	265,000	17,450	81	25	2.3	
**6**	7.6	84,000	19,830	65	21		128
**8**	6.6	108,000	21,850	48	15.4	2.1	133
**9**	7.2	143,000	33,320	53	13.9		
**11**	6.7	298,000	47,100	41	14.6	2.2	
**17**	7.9	850,000	35,600	43	6.7	2.5	
**21**	8.2	1,230,000	32,000	48	5.1	2.6	
**26**	8.8	950,000	28,300	42	3.2	2.8	
**37**	9.8	680,000	23,000	39	0.2	2.5	

Finally, we used clopidogrel (1mg/Kg/day) instead of acetylsalicylic acid at low dose for its anti-aggregant effects because of the persistence of the ectasia and the dilatation of the coronary arteries (the maximum diameters were 0.71 cm for RCA, 0.42 cm for LCA and 0.4 cm for the left anterior descending artery - LAD).

## Discussion

KD is commonly a self-limiting vasculitis although coronary artery aneurysms may occur in approximately 25-30% of untreated patients. This complication represents the most important adverse prognostic factor and it is the leading cause of death for acquired heart disease in children [6,10]. Coronary abnormalities occur more frequently during the sub-acute phase of the disease and Harada [11] and Beiser [12] scores represent the most predictive risk index to identify this complication.

A timely diagnosis and an early beginning of treatment represent a key clinical skill to prevent cardiac complications [13-15].

In our case, 6 days after the onset of fever we detected an early alteration in the wall of both RCA and LAD and during the following days an increase in RCA diameter (0.41 cm).

Usually KD shows the highest incidence in children under five years of age whereas is very rare under three months of age (1.6%) [16,17].

The etiology of KD remains unknown, however a combination of microbial infection and the immune response or genetic susceptibility are believed to contribute to the development of KD, as has been suggested for atopic diseases [18] and much attention has focused on the role of a variety of genes related to inflammation [19].

Moreover the clinical and epidemiological features of KD also suggest that infectious agents might trigger the development of this disease [20], although no specific pathogens have been identified and significant contribution of the innate immune system to the pathophysiology of the acute phase of the disease has been demonstrated in recent studies [21].

Six-month-old patients or younger often show incomplete clinical features and this phenotype is linked with a higher percentage of coronary artery anomalies [13,16,22-24].

According to standard therapy, 80-90% of treated patients show a clinical and biochemical remission; in the remaining percentage of patients a persistent fever represents a sign of unresponsiveness to IVIG which is the major risk factor for the development of coronary artery lesions [7].

Kobayashi [7], Egami [8] and Sano [9] proposed three different scoring systems in order to identify the patients at high risk for unresponsiveness to therapy with IVIG. According to these scores, in our patient the value resulted higher than the cut-off for each one.

The use of IVMP and its efficacy in refractory forms is still unclear. According to some authors, the use of IVMP seems to be associated with a decrease of fever. It remains uncertain its role on the progression of coronary artery anomalies although some other authors suggested a preventive role if IVMP is used during an early phase of the disease [25-27].

Our patient’s clinical evolution seems to show that before three months of age KD is associated with a high risk of severe presentation and unresponsiveness to therapy. In fact, in our patient an early diagnosis and a timely starting of IVIG therapy was ineffective in preventing coronary artery aneurysms.

The key to prevent this dangerous cardiac involvement is still unknown although the resolution of systemic inflammation as early as possible seems to represent the target of the therapy [27]. In this regard, in the absence of a standardized regime for resistant forms with a high risk of development of coronary artery anomalies, we suggest considering IVMP associated with IVIG even during an early phase of the disease if the score indexes are predictive.

Furthermore since inflammatory and immunologic processes of the innate immune system seem to have a pivotal role in the development of KD even infliximab administration or plasma exchange can be very important rescue methods for children refractory to IVIG [28].

## Conclusion

Our case is notable because of the very young age of the patient, the severity of clinical presentation with an early development of coronary artery aneurysms and the unresponsiveness to the therapy.

## Consent

Written informed consent was obtained from the parents of the patient for publication of this case report and accompanying images. A copy of the written consent is available for review by the editor-in-Chief of this journal.

## Abbreviations

KD: Kawasaki disease; IVIG: Intravenous immunoglobulin; IVMP: Intravenous methylprednisolone; RCA: Right coronary artery; LCA: Left coronary artery; LAD: Left anterior descending artery.

## Competing interests

The authors declare that they have no competing interests.

## Authors’ contributions

All the authors participated sufficiently in preparation of this manuscript SL, PB, GG, VD–followed the patient in his clinical course. PS performed cardiovascular examinations. GG and GFP–revised the literature. GFP and SL–took care of the revisions of the Ms. SL, ML–made the final analysis and critical revision of this Ms. All authors gave final approval for manuscript publication.
